# Ethnic differences in the association of *SERPING1* with age-related macular degeneration and polypoidal choroidal vasculopathy

**DOI:** 10.1038/srep09424

**Published:** 2015-03-24

**Authors:** Ke Liu, Timothy Y. Y. Lai, Li Ma, Frank H. P. Lai, Alvin L. Young, Marten E. Brelen, Pancy O. S. Tam, Chi Pui Pang, Li Jia Chen

**Affiliations:** 1 Department of Ophthalmology and Visual Sciences, the Chinese University of Hong Kong, Hong Kong, China; 2 Shenzhen Eye Hospital, Shenzhen, China; 3 Department of Ophthalmology and Visual Sciences, Prince of Wales Hospital, the Chinese University of Hong Kong, Hong Kong, China; 4 Shenzhen Key Laboratory of Ophthalmology, Shenzhen, China

## Abstract

Neovascular age-related macular degeneration (AMD) and polypoidal choroidal vasculopathy (PCV) are leading causes of irreversible blindness in developed countries. In this study, we investigated the association of single nucleotide polymorphisms (SNPs) in the *serpin peptidase inhibitor, clade G, member 1* (*SERPING1*) gene with neovascular AMD and PCV. Two haplotype-tagging SNPs, rs1005510 and rs11603020, of *SERPING1* were genotyped in 708 unrelated Chinese individuals: 200 neovascular AMD, 233 PCV and 275 controls. A meta-analysis was also performed for all reported associations of *SERPING1* SNPs with AMD and PCV. None of the tagging SNPs had a significant association with neovascular AMD or PCV (*P* > 0.05) in our study cohort. The meta-analyses showed that the most-studied SNP rs2511989 was not significantly associated with all forms of AMD, neovascular AMD, or PCV in East Asians (*P* = 0.98, 0.93 and 0.30, respectively) but was associated with AMD in Caucasians (*P* = 0.04 for all AMD and 0.004 for neovascular AMD). Therefore, the results of our study and meta-analysis suggest that *SERPING1* is not a major genetic component of AMD or PCV in East Asians but is a genetic risk factor for AMD in Caucasians, providing evidence for an ethnic diversity in the genetic etiology of AMD.

Age-related macular degeneration (AMD) is a leading cause of irreversible visual loss among the elderly population worldwide and is accounting for approximately 8.7% of all blindness due to eye disease^1^. In its early stages, AMD has a slow and insidious onset often taking many years to progress^2^. In the advanced stages, geographic atrophy or neovascular AMD can lead to acute and rapid visual loss. Therefore, early detection of individuals who are at risk of progression is essential. AMD has a complex etiology, and the major risk factors include older age, cigarette smoking and genetic susceptibility^2^,^3^. To date, single-nucleotide polymorphisms (SNPs) in over 20 genetic loci have been associated with AMD. Among them, the *complement factor H* gene (*CFH*) gene and the *ARMS2-HTRA1* locus show the highest effect sizes and have been confirmed in different populations^4^,^5^,^6^,^7^.

Polypoidal choroidal vasculopathy (PCV), which is considered a subtype of neovascular AMD, is characterized by polypoidal dilatations of the inner choroidal vascular network. The polyps can be visualized with indocyanine green angiography (ICGA). Patients present with persistent and recurrent serous leakage and occasionally hemorrhage at the macula^8^. The frequency of PCV among neovascular AMD patients in East Asians (Korean, Japanese and Chinese) ranged from 24.5% to 54.7%which is much higher than that in Caucasians (4%)^9^,^10^,^11^,^12^,^13^. There are likely genetic and environmental factors that underlie the pathogenesis of PCV that explains the different prevalence in different populations^14^,^15^,^16^. AMD susceptibility genes, including *CFH* and *ARMS2*-*HTRA1*, have been investigated in PCV but their associations were variable in different populations^17^,^18^,^19^,^20^.

Recently, Ennis *et al* identified a significant association between an intronic SNP rs2511989 in the *complement component 1 inhibitor* gene (also known as *serpin peptidase inhibitor, clade G, member 1*, *SERPING1*) and AMD in the Caucasian population^21^. However, findings in subsequent studies on the genetic association of *SERPING1* with AMD in other populations were inconsistent^22^,^23^,^24^. Among subtypes of AMD, *SERPING1* was associated with neovascular AMD^25^ but not with PCV^26^,^27^. Therefore, investigation of *SERPING1* in AMD and PCV in more study cohorts and a meta-analysis of reported associations are warranted to confirm the role of *SERPING1* in these diseases.

In this study, we conducted a haplotype-tagging SNP association analysis in a Chinese cohort to evaluate the role of *SERPING1* in AMD and PCV. A meta-analysis of all reported *SERPING1* SNPs in AMD and PCV was also performed.

## Results

In this study, a total of 708 unrelated study subjects were enrolled, comprising 200 patients with neovascular AMD, 233 with PCV and 275 controls (Table 1). Since there were more males recruited in the disease groups compared to the controls, the association analysis was adjusted for gender using logistic regression. We purposely recruited subjects older than 60 years as controls for late-onset diseases therefore the mean age of the control group was greater than that of the PCV group.

### Individual single nucleotide polymorphism analysis

Two haplotype-tagging SNPs, rs1005510 and rs11603020, which capture all alleles across the *SERPING1* locus with a minor allele frequency larger than 0.1 and a mean r^2^ of 1.00 in the HapMap CHB population, were genotyped in all study subjects with a call rate of 99.6%. The distribution of the genotypes in all study groups followed Hardy-Weinberg equilibrium (HWE) (*P* > 0.05). In association analysis, we found these two SNPs were not significantly associated with neovascular AMD or PCV in allelic, dominant or recessive models (*P* > 0.05, Table 2). No significant association was found after adjusted for gender and age in logistic regression (data not shown). Also, the two SNPs showed no significant difference between neovascular AMD and PCV (Table 2).

### Linkage disequilibrium and haplotype analysis

Linkage disequilibrium (LD) analysis showed that the 2 tagging SNPs were included in one haplotype block in both neovascular AMD and PCV. Comparison of the haplotypes in neovascular AMD and PCV showed a similar distribution of haplotypes. No haplotype was significantly associated with neovascular AMD or PCV (*P* > 0.1, Table 3).

### Interaction of *SERPING1* SNPs with other AMD/PCV genes and gender

Epistasis analysis identified no significant gene-gene interaction between the 2 *SERPING1* SNPs and the 2 major susceptibility variants for AMD and PCV, *CFH* rs800292 and *HTRA1* rs11200638 (*P* > 0.05), data of which were obtained from our previous studies^14^,^15^. Also, no SNP-gender interaction was identified for the 2 *SERPING1* SNPs. In the full epistatic models, the *SERPING1* SNPs were not associated with AMD or PCV when conditioned on rs800292, rs11200638, gender and the interaction terms (data not shown).

### Meta-analysis of *SERPING1* variants in AMD and PCV

We performed a meta-analysis of the associations of all reported *SERPING1* SNPs with AMD and PCV. A total of 56 articles were identified from literature search on May 29, 2014, including 28 from PubMed and 28 from Embase. Among them, 43 were excluded, including 22 duplications, 15 with unrelated topics, 3 reviews, and 3 comments. The remaining 13 reports were thoroughly reviewed and 4 of them were further excluded because the study cohorts in 3 were duplicated with others and 1 was not an association study (Supplementary Fig. S1). We also manually searched in the texts and supplementary materials of all reported genome-wide association studies (GWAS) of AMD and found 4 relevant studies^27^,^28^,^29^,^30^. However, samples in two studies^27^,^28^ were included in a later study with a larger sample size^29^. Therefore, only the latest study was included for meta-analysis^29^. Finally, 11 studies were included in the meta-analysis^21^,^22^,^23^,^24^,^25^,^26^,^27^,^29^,^30^,^31^,^32^. Totally 15 SNPs in *SERPING1* had been reported in AMD and 4 SNPs in PCV. However, only 4 SNPs (rs2511989, rs1005510, rs2511990 and rs11603020) in AMD and 1 SNP (rs2511989) in PCV were included for meta-analyses. These SNPs had genetic data available in at least two independent studies.

**Figure 1:**
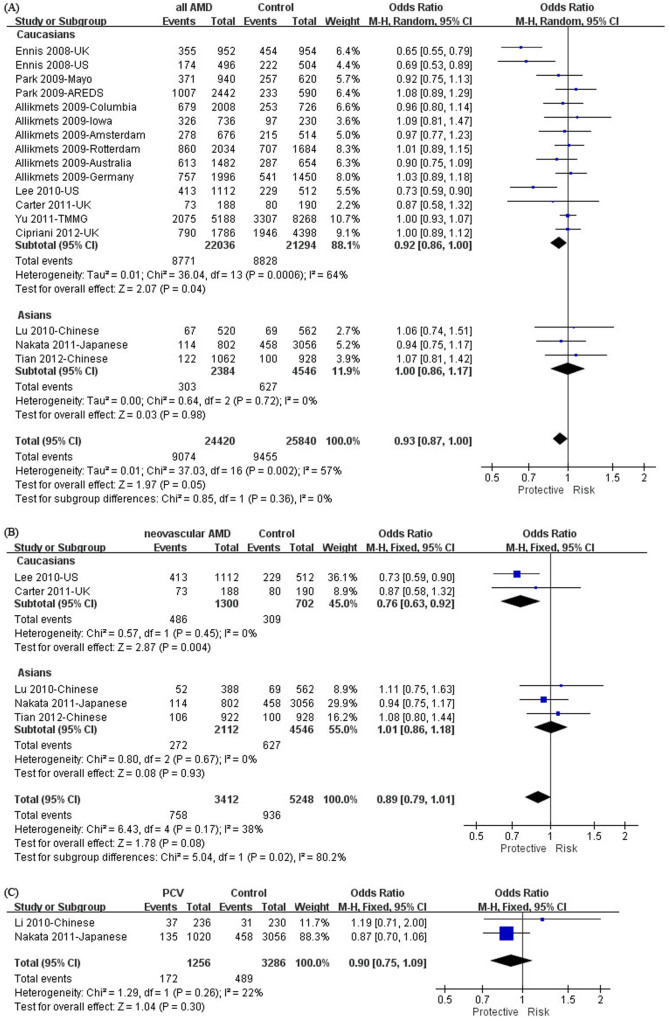
Forest plots of meta-analysis for rs2511989 in (A) all forms of AMD, (B) neovascular AMD, and (C) PCV. Individual and pooled odds ratios (OR) were estimated for the A allele. Squares indicate the study-specific OR. The size of the box is proportional to the percent weight that each study contributed in the pooled OR. Horizontal lines indicate 95% confidence intervals (CI). A diamond indicates the pooled OR with 95% CI. “Events” mean the counts of the A allele. “Total” means the total allele counts in cases or controls. “Weight” indicates how much an individual study contributes to the pooled estimate. “H-M” stands for the Mantel-Haenszel method in meta-analysis. “Random” indicates that a random-effects method was adopted for generating the meta-analysis result. AMD: age-related macular degeneration; PCV: polypoidal choroidal vasculopathy.

SNP rs2511989 is the most commonly investigated in *SERPING1*. It was involved in 17 independent case-control cohorts reported in 10 studies^21^,^22^,^23^,^24^,^25^,^26^,^29^,^30^,^31^,^32^, involving 11018 cases and 10647 controls. Meta-analysis showed that the association of rs2511989 with all forms of AMD was marginal (*P* = 0.05, odds ratio (OR) = 0.93, 95% confidence interval (CI): 0.87–1.00, *I*^*2*^ = 57%; Figure 1A). Subgroup analysis by ethnicity revealed that this SNP was associated with all AMD in Caucasians (*P* = 0.04, OR = 0.92, 95% CI: 0.86–1.00, *I*^*2*^ = 64%; Figure 1A) but not in East Asians (*P* = 0.98, OR = 1.00; 95% CI: 0.86–1.17, *I*^*2*^ = 0%; Figure 1A). In the sensitivity analysis in Caucasians, the pooled allelic ORs were not statistically significant when we excluded, each at a time, the cohorts in the study of Ennis *et al*^21^ (UK cohort excluded: *P* = 0.16, OR = 0.96, 95% CI: 0.90–1.02, *I*^*2*^ = 36%; US cohort excluded: *P* = 0.09, OR = 0.94, 95% CI: 0.87–1.01, *I*^*2*^ = 59%) or Lee *et al*^25^ (*P* = 0.10, OR = 0.94, 95% CI: 0.87–1.01, *I*^*2*^ = 60%). In contrast, the pooled ORs remained significant when we excluded any one of the other studies (*P* values: 0.02–0.05). In neovascular AMD, rs2511989 also showed a significant association in Caucasians (*P* = 0.004, OR = 0.76, 95% CI: 0.63–0.92, *I*^*2*^ = 0%), but not in Asians (*P* = 0.93, OR = 1.01, 95% CI: 0.86–1.18, *I*^*2*^ = 0%; Figure 1B). When all study populations were pooled, the association was not significant (*P* = 0.08, OR = 0.89, 95% CI: 0.79–1.01, *I*^*2*^ = 38%; Figure 1B). Sensitivity analysis was not done in Caucasians as there were only two studies included in the meta-analysis. In these studies, the effects of the SNP were toward the same direction (Figure 1B). In PCV, rs2511989 did not show a significant association in Asians (*P* = 0.30, OR = 0.90, 95% CI: 0.75–1.09, *I*^*2*^ = 22%; Figure 1C), whereas there is no data from Caucasians.

**Figure 2:**
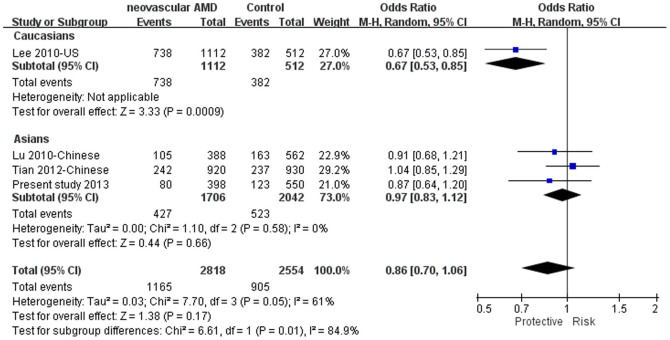
The forest plots of meta-analysis for rs1005510(G) in neovascular AMD. Squares indicate study-specific odds ratios (ORs). The size of the box is proportional to the percent weight that each study contributed in the pooled OR. Horizontal lines indicate 95% confidence intervals (CI). A diamond indicates the pooled OR with 95% CI. “Events” mean the counts of the G allele. “Total” means the total allele counts in cases or controls. “Weight” indicates how much an individual study contributes to the pooled estimate. “H-M” stands for the Mantel-Haenszel method in meta-analysis. “Random” indicates that a random-effects method was adopted for generating the meta-analysis result. AMD: age-related macular degeneration.

Genotype data of SNP rs1005510 was available from 3 studies on all forms of AMD and neovascular AMD^24^,^25^,^31^, and from 1 study on PCV^27^. Meta-analysis showed that this SNP was not significantly associated with neovascular AMD in the pooled population including our sample (*P* = 0.17; OR = 0.86, 95% CI: 0.70–1.06, *I*^*2*^ = 61%; Figure 2). Notably, rs1005510 was involved in only 1 Caucasian study, in which a significant association was detected (*P* = 0.0009, OR = 0.67, 95% CI: 0.53–0.85)^25^. In East Asians no significant association was detected in the pooled population (*P* = 0.66, OR = 0.97, 9%% CI: 0.83–1.12, *I*^*2*^ = 0%; Figure 2). Of note, all of the 3 Asian studies involved Chinese subjects. The odds ratio in the study of Tian et al. was toward a different trend.

**Figure 3:**
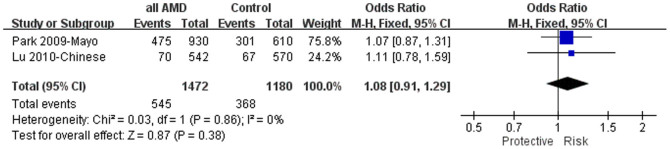
The forest plot of meta-analysis for rs2511990(T) in AMD with all subtypes. Squares indicate study-specific odds ratios (ORs). The size of the box is proportional to the percent weight that each study contributed in the pooled OR. Horizontal lines indicate 95% confidence intervals (CI). A diamond indicates the pooled OR with 95% CI. “Events” mean the counts of the T allele. “Total” means the total allele counts in cases or controls. “Weight” indicates how much an individual study contributes to the pooled estimate. “H-M” stands for the Mantel-Haenszel method in meta-analysis. “Fixed” indicates that a fixed-effects method was adopted for generating the meta-analysis result. AMD: age-related macular degeneration.

**Figure 4:**
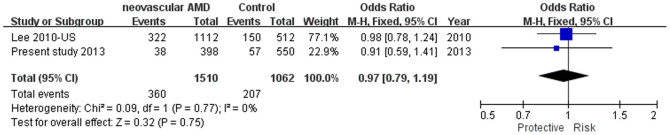
The forest plot of meta-analysis for rs11603020(C) in neovascular AMD. Squares indicate study-specific odds ratios (ORs). The size of the box is proportional to the percent weight that each study contributed in the pooled OR. Horizontal lines indicate 95% confidence intervals (CI). A diamond indicates the pooled OR with 95% CI. “Events” mean the counts of the C allele. “Total” means the total allele counts in cases or controls. “Weight” indicates how much an individual study contributes to the pooled estimate. “H-M” stands for the Mantel-Haenszel method in meta-analysis. “Fixed” indicates that a fixed-effects method was adopted for generating the meta-analysis result. AMD: age-related macular degeneration.

SNP rs2511990 was reported in 2 studies^22^,^31^, it was not associated AMD (*P* = 0.38; OR = 1.08, 95% CI: 0.91–1.29, *I*^*2*^ = 0%; Figure 3). Also, SNP rs11603020 was not associated with neovascular AMD in a Caucasian population^25^. When combined with the data of our present study, there was still no significant association between rs11603020 and neovascular AMD, although the odds ratios were toward the same trend in the both cohorts (*P* = 0.75, OR = 0.97, 95% CI: 0.79–1.19, *I*^*2*^ = 0%; Figure 4).

## Discussion

In this study, we performed a haplotype-tagging SNP analysis of the *SERPING1* gene in neovascular AMD and PCV, and a meta-analysis of reported *SERPING1* SNPs in the two diseases. We found that none of the SNPs or haplotypes was significantly associated with AMD or PCV in Chinese. Moreover, the *SERPING1* SNPs have no statistical interaction with SNPs in the two major AMD genes, *CFH* and *HTRA1*. Furthermore, the meta-analysis revealed that the common SNPs rs2511989, rs1005510, rs2511990 and rs11603020 in *SERPING1* were not significantly associated with all forms of AMD or neovascular AMD in Japanese and Chinese, and the SNP rs2511989 was not associated with PCV. In contrast, rs2511989 was significantly associated with AMD (especially neovascular AMD) in Caucasians. These findings together suggest that *SERPING1* is not a disease gene for AMD or PCV in East Asians, but is likely to be a susceptibility gene for AMD in Caucasians.

SERPING1 inhibits the activation of the classical and lectin complement pathways by suppressing the activity of complement component 1 and mannan-binding lectin serine peptidase 2^22^,^33^,^34^. Because of its important regulatory role in the complement pathway, *SERPING1* was considered a candidate gene for AMD. Ennis *et al* first identified a significant association between *SERPING1* rs2511989 and all subtypes of AMD in two independent cohorts^21^. Later, Lee *et al* showed that *SERPING1* rs2511989 and rs1005510 were significantly associated with neovascular AMD^25^. However, significant association between *SERPING1* and AMD was not found in others studies^22^,^23^,^24^,^26^,^31^,^32^, suggesting ethnic diversity. By comparing the LD structures of the *SERPING1* locus among the CEU, CHB and JPT HapMap populations (Supplementary Fig. S2), we found that the three populations have similar LD patterns. Therefore, the discrepancies in the association profiles between Caucasians and Asians are more likely due to population-specific effects rather than difference in the LD structures.

In fact, ethnic diversities are also seen in other AMD genes, such as *CFH*. In Caucasians, the minor allele frequency (MAF) of the *CFH* SNP rs1061170 is greater than 20% and it was associated with AMD even in small cohorts^4^. In Asians, rs1061170 has a MAF of less than 10% and was not significantly associated with AMD in separate study cohorts^35^,^36^. However, when being meta-analyzed, rs1061170 showed a significant association with AMD in Asians^37^. Thus, the inconsistent association profiles in different studies may be due to insufficient statistical power in individual studies or population-specific effects. Thus, a meta-analysis with stratification analysis by ethnicity could increase the statistical power for detecting the association in the pooled study subjects and specific ethnic groups. Moreover, the main effect of the gene could be modified by other factors, such as by gene-gene or gene-environmental interactions. In this study, we found no gene-gene or gene-gender interaction for *SERPING1*.

In this study we did not include rs2511989 because its MAF is less than 0.1 in Chinese and was not picked by the tagger program in HapMap. Indeed, the MAF of rs2511989 in Chinese is much lower than that in Caucasians (0.425) and Japanese (0.174) according to the HapMap database, thus the statistical power for detecting a significant association would be less than 40% in our study, assuming an α level of 0.05 and an odds ratio of 0.67 according to the study of Ennis *et al*^21^. In the meta-analysis we found that rs2511989 was not significantly associated with all forms of AMD in Japanese and Chinese (1192 cases versus 2273 controls) or neovascular AMD (1056 cases versus 2273 controls). Such sample sizes provided over 95% statistical power to detect a significant association for all forms of AMD (OR = 0.67^21^) or neovascular AMD (OR = 0.73^25^). Therefore, rs2511989 is not an associated factor for AMD in East Asians. Also, rs2511989 is not associated with PCV in East Asians (Figure 1C). Of note, a previous meta-analysis had shown that rs2511989 was not associated with PCV^19^. However, in that meta-analysis, only 1 control group was included from the study of Nakata^26^, whilst in our present meta-analysis, both of the control groups^26^ were included and the results remained consistent.

The present study provides a comprehensive evaluation of the *SERPING1* gene in AMD and PCV, using haplotype-tagging SNP analysis and meta-analysis. Though the data is robust, there are several limitations. First, the gender ratios were not matched in the study groups. This discrepancy was due to the fact that the patients were recruited consecutively in the clinics and the gender ratio was not purposely confined. Therefore, we corrected the gender imbalance by logistic regression and found it had no impact to the association results. Second, the smoking status for a portion of our study subjects was not available so that smoking was not adjusted in the analysis. Third, in the meta-analysis of rs2511989 in Caucasians, although the association was significant in the pooled populations, there was significant inter-cohort heterogeneity (*I*^*2*^ = 64%). In the sensitivity analysis, we found that the associations were not significant after we removed each of the 3 cohorts included in the studies of Ennis *et al* and Lee *et al*^21^,^25^; thus the association result could have been dominated by these studies. Further studies in larger samples of matched ethnic backgrounds are warranted to confirm the role of *SERPING1* in AMD among Caucasians.

In summary, this study showed that the tagging SNPs rs1005510 and rs11603020 in *SERPING1* are not associated with neovascular AMD or PCV in Chinese. Also, our meta-analysis revealed that these 2 SNPs were not implicated in AMD among Japanese and Chinese. Therefore, *SERPING1* is less likely to be major gene for neovascular AMD or PCV in East Asians. In contrast, our meta-analysis suggests that SNPs rs2511989 and rs1005510 in *SERPING1* could be genetic markers for AMD in Caucasians, indicating an ethnic difference.

## Methods

### Study participants

The study protocol was approved by the Ethics Committee on Human Research, the Chinese University of Hong Kong. The study procedures were performed in accordance with the tenets of the Declaration of Helsinki. Written informed consent was obtained from every subject. All study subjects were Han Chinese recruited from the Hong Kong Eye Hospital and the Eye Centre of the Prince of Wales Hospital, Hong Kong.

All patients received complete ophthalmic examinations, including best-corrected visual acuity measurement, slit-lamp biomicroscopy, color fundus photographs, fluorescein angiography, and ICGA. All AMD patients had neovascular AMD in at least one eye. PCV was diagnosed by ICGA showing a choroidal origin of polypoidal lesions. Patients with CNV due to other causes, such as myopic maculopathy, or with both CNV and PCV lesions in the same or fellow eye, were excluded. Unrelated control subjects were recruited from people who attended the clinic for eye examinations and included on the following criteria: (1) age >60 years; (2) no age-related maculopathy or macular degeneration; and (3) no any other major eye diseases, except for mild senile cataracts and mild refractive errors. The characteristics of the study subjects are summarized in Table 1.

### SNP selection and genotyping

We adopted a haplotype-tagging SNP approach and obtained the tagging SNPs across the *SERPING1* region with r^2^ and minor allele frequency greater than 0.8 and 0.10, respectively, from the International HapMap Project for the Chinese Han Beijing (CHB) population (http://hapmap.ncbi.nlm.nih.gov/, HapMap Genome Browser, accessed Jun 20, 2013). Two tagging SNPs, rs1005510 and rs11603020, were identified by the tagger-pairwise method. These two SNPs captured 6 common SNPs, including rs2511988, rs1005510 and rs1005511 captured by rs1005510, and rs3824988, rs11229067 and rs11603020 captured by rs11603020.

Genomic DNA was extracted from whole blood using a QIAamp Blood Kit (Qiagen, Hilden, Germany) according to the protocol from the manufacturer. SNPs rs1005510 and rs11603020 were genotyped using *TaqMan* genotyping assays (Applied Biosystems [ABI], Foster City, CA) on a Roche LightCycler® 480 Real-Time PCR System (Roche, Switzerland) according to the manufacturer's instructions.

### Statistical analysis

Hardy-Weinberg Equilibrium (HWE) of individual SNP in the case and control groups were tested using the exact test implemented in the software package PLINK (v1.07, http://pngu.mgh.harvard.edu/purcell/plink/)^38^. Allelic and genotypic associations of both SNPs with neovascular AMD and PCV were evaluated using the chi-square test or Fisher's exact test in PLINK, with or without adjustment for gender and age. The odds ratio and corresponding 95% confidence interval were estimated with the major allele as reference. Pairwise linkage disequilibrium and haplotype associations were assessed using the Haploview software^39^. Haplotype blocks were determined using the confidence interval method in Haploview. The epistasis algorithm in PLINK was applied to detect gene-gene interaction between the two *SERPING1* SNPs and two major gene variants for AMD, *CFH* rs800292 and *HTRA1* rs11200638. Genotype data of the latter two SNPs were obtained from our previous study^14^. Also, interaction between the *SERPING1* SNPs and gender was analyzed using logistic regression (SPSS ver.20.0, SPSS Inc., Chicago, IL).

### Meta-analysis

A systematic literature search in the PubMed and Embase databases was conducted on May 29, 2014 to identify published studies on the association of *SERPING1* with AMD and/or PCV. All publications in English language between January 1, 2005 and May 29, 2014 were included. The searching term “(age related macular degeneration OR AMD OR polypoidal choroidal vasculopathy OR PCV) AND (SERPING1 OR C1 inhibitor OR C1IN OR C1INH OR C1NH)” in all fields was used. We also manually screened the reference lists of all eligible articles. Moreover, to maximize the usable data we searched all reported genome-wide association studies of AMD including their supplementary materials. Two reviewers (K.L. and L.M.) independently reviewed the retrieved records. Any discrepancies were resolved by another reviewer (L.J.C.) after thorough discussion. The inclusion criteria were: 1) case-control association studies of *SERPING1* variants with AMD and PCV; 2) raw data of allele or genotype counts available; and 3) the type of article being an original research study but not a review, case report or comment. For studies published by the same group on the same SNPs, only the most recent study or the study with the largest sample size was used; while for SNPs in linkage disequilibrium (LD), only the most commonly investigated one was selected for the meta-analysis. The following information was extracted from each record: the name of first author, publication year, ethnicity of the study population, study design, sample size, disease subtype, gender composition, mean age, allele and genotype distribution in cases and controls, Hardy-Weinberg equilibrium (HWE) test results in controls. Allelic associations of the SNPs in different studies were meta-analyzed and the pooled odds ratio and 95% confidence interval were estimated for the strength of association, using the fixed-effect (*I*^*2*^ ≤ 50%) or random-effect (*I*^*2*^ > 50%) model based on the heterogeneity test^40^. The *I*^*2*^ test was used to assess heterogeneity among studies. The *I*^*2*^ value was explained as of no (0–25%), low (25–50%), moderate (50–75%) and high heterogeneity (75–100%)^41^. Sensitivity analysis was also conducted to examine the potential effect of a study by removing the studies each at a time. All meta-analyses were conducted using the software Review Manager (RevMan, version 5.2, The Cochrane Collaboration, Copenhagen, Denmark). A pooled *P* value of less than 0.05 was considered statistically significant.

## Author Contributions

K.L. and L.J.C. designed the experiments. K.L., L.M. and P.T. performed the experiments. K.L. and L.M. performed the data analysis and meta-analysis. K.L. wrote the paper. T.L., F.L. and A.Y. contributed the clinical samples. T.L., M.B., C.P.P. and L.J.C. revised the paper. M.B. edited the English style. All authors contributed to scientific discussions.

## Supplementary Material

Supplementary Information Supplementary material

## Figures and Tables

**Table 1:** Characteristics of the study subjects

	AMD (n = 200)	PCV (n = 233)	Control (n = 275)	Comparison (*P* value)	
				AMD-Control	PCV-Control
Gender (male/female)	110/90	162/71	121/154	0.02	<0.05
Mean age ± SD (years)	75.3 ± 7.7	68.5 ± 9.0	74.3 ± 7.6	0.16	<0.05
Age range (years)	50–94	43–90	60–94	/	/

AMD: age related macular degeneration; PCV: polypoidal choroidal vasculopathy; SD: standard deviation.

**Table 2:** Association of haplotype-tagging SNPs of *SERPING1* with neovascular AMD and PCV

		Allelic Distribution (%)			Allelic Association (*P* value) Odds Ratio (95% Confidence Intervals)				Genotype Distribution (%)			Genotypic Association (*P* value)	
SNP ID		AMD	PCV	Control	AMD-Control	PCV-Control	PCV-AMD		AMD (n = 200)	PCV (n = 233)	Control (n = 275)	AMD-Control	PCV-Control
rs1005510	G	80 (20.10)	99 (21.43)	123 (22.36)	0.40	0.72	0.63	GG	10 (5.03)	11 (4.76)	15 (5.45)	0.67^a^	0.94^a^
	A	318 (79.90)	363 (78.57)	427 (77.64)	0.87 (0.64–1.2)	0.95 (0.70–1.28)	1.08 (0.78–1.51)	AG	60 (30.15)	77 (33.33)	93 (33.82)	0.39^b^	0.85^b^
								AA	129 (64.82)	143 (61.91)	167 (60.73)	1.00^c^	0.84^c^
rs11603020	C	38 (9.55)	45 (9.74)	57 (10.36)	0.68	0.74	0.92	CC	1 (0.50)	1 (0.43)	2 (0.73)	0.92^a^	0.96^a^
	T	360 (90.45)	417 (90.26)	493 (89.64)	0.91 (0.59–1.41)	0.93 (0.62–1.41)	1.02 (0.65–1.61)	CT	36 (18.09)	43 (18.62)	53 (19.27)	0.73^b^	0.82^b^
								TT	162 (81.41)	187 (80.95)	220 (80.00)	1.00^c^	1.00^c^

^a^genotypic association;

^b^association in the dominant model;

^c^association in the recessive model;

AMD: age related macular degeneration; PCV: polypoidal choroidal vasculopathy; SNP: single nucleotide polymorphism.

**Table 3:** Haplotype association of *SERPING1* with neovascular AMD and PCV

Haplotype	Frequency			Association (*P* value)	
rs1005510- rs11603020	AMD	PCV	Control	AMD-Control	PCV-Control
A-T	0.79	0.78	0.77	0.47	0.86
G-T	0.11	0.12	0.12	0.59	0.94
G-C	0.09	0.09	0.10	0.56	0.56

AMD: age related macular degeneration; PCV: polypoidal choroidal vasculopathy.
