# Supplementary material for: Ethnic differences in the association of SERPING1 with age-related macular degeneration and polypoidal choroidal vasculopathy
Source: Sci Rep. 2015 Mar 24;5:9424. doi: 10.1038/srep09424 (PMC4371106; doi:10.1038/srep09424)
Supplement: Supplementary Information — Supplementary material [file srep09424-s1.pdf]

Ethnic differences in the association of *SERPING1* with age-related macular degeneration and polypoidal choroidal vasculopathy

Ke Liu,[1,2] Timothy Y.Y. Lai,[1] Li Ma,[1] Frank H.P. Lai,[3] Alvin L. Young,[1,3] Marten E. Brelen,[1,3] Pancy O.S. Tam,[1] Chi Pui Pang,[1,3] Li Jia Chen.[1,3]

[1]Department of Ophthalmology and Visual Sciences, the Chinese University of Hong Kong, Hong Kong, China; [2]Shenzhen Eye Hospital, Shenzhen, China. [3]Department of Ophthalmology and Visual Sciences, Prince of Wales Hospital, the Chinese University of Hong Kong, Hong Kong, China.

Correspondence:
Dr Li Jia Chen
Department of Ophthalmology and Visual Sciences, The Chinese University of Hong Kong, Hong Kong Eye Hospital, 147K Argyle Street, Kowloon, Hong Kong.
Tel: +852 39435810; Fax: +852 27159490; E-mail: lijia_chen@cuhk.edu.hk

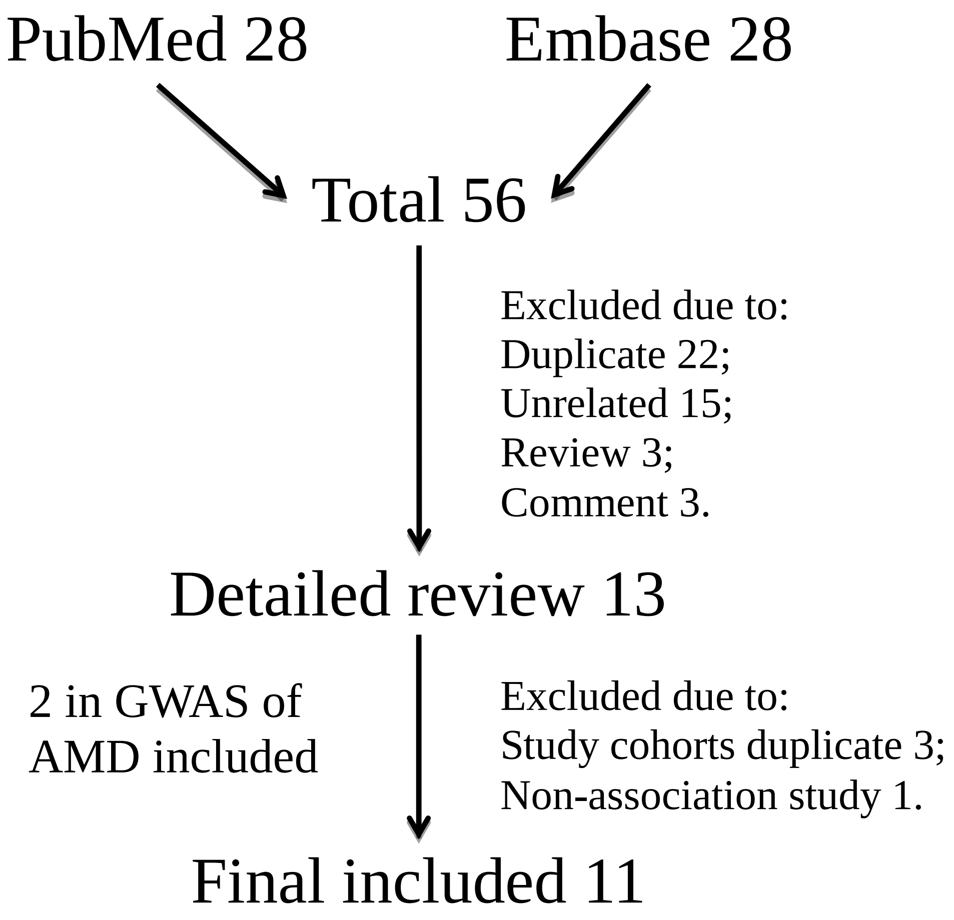

Supplementary Fig S1. Flow diagram of literature screening. The flow diagram showed the screening process of retrieved articles, including the number and reason of exclusion.

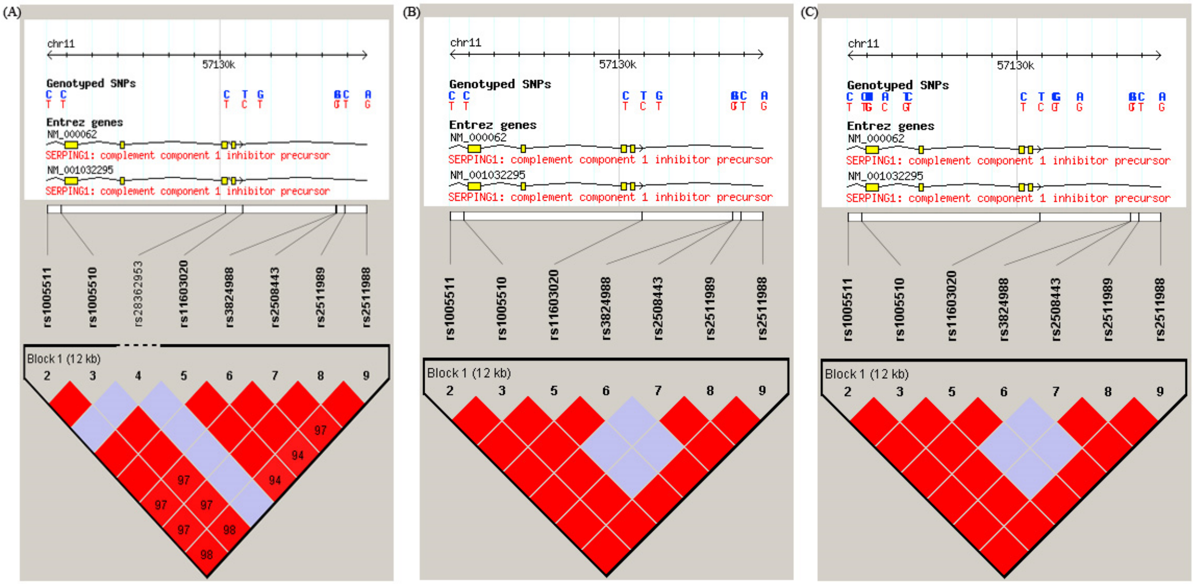

Supplementary Fig S2. Linkage disequilibrium (LD) structures of *SERPING1* across the CEU (A), CHB (B) and JPT (C) HapMap populations. The haplotype block was defined by the confidence interval method implemented in the Haploview software. The LD (r^2) was listed in the cross cell between any two of SNPs. CEU: Utah residents with Northern and Western European ancestry from the CEPH collection; CHB: Han Chinese in Beijing, China; JPT: Japanese in Tokyo, Japan.
